# Very small embryonic-like stem cells are involved in pancreatic regeneration and their dysfunction with age may lead to diabetes and cancer

**DOI:** 10.1186/s13287-015-0084-3

**Published:** 2015-05-15

**Authors:** Deepa Bhartiya, Hiren Patel

**Affiliations:** Stem Cell Biology Department, National Institute for Research in Reproductive Health, JM Street, Parel, Mumbai, 400012 India

## Abstract

Mouse pancreas has a remarkable ability to regenerate after partial pancreatectomy, and several investigators have studied the underlying mechanisms involved in this regeneration process; however, the field remains contentious. Elegant lineage-tracing studies undertaken over a decade have generated strong evidence against neogenesis from stem cells and in favor of reduplication of pre-existing islets. Ductal epithelium has also been implicated during regeneration. We recently provided direct evidence for the possible involvement of very small embryonic-like stem cells (VSELs) during regeneration after partial pancreatectomy in mice. VSELs were first reported in pancreas in 2008 and are mobilized in large numbers after treating mice with streptozotocin and in patients with pancreatic cancer. VSELs can be detected in mouse pancreas as small-sized LIN^−^/CD45^−^/SCA-1^+^ cells (3 to 5 μm), present in small numbers (0.6%), which express nuclear Oct-4 (octamer-binding transcription factor 4) and other pluripotent markers along with their immediate descendant ‘progenitors’, which are slightly bigger and co-express Oct-4 and PDX-1. VSELs and the progenitors get mobilized in large numbers after partial pancreatectomy and regenerate both pancreatic islets and acinar cells. In this review, we deliberate upon possible reasons why VSELs have eluded scientists so far. Because of their small size, VSELs are probably unknowingly and inadvertently discarded during processing. Similar to menopause and related loss of ovarian function, type 2 diabetes mellitus occurs because of a decline in beta-cell function possibly resulting from an age-related compromised niche which does not allow VSELs to maintain normal homeostasis. As suggested earlier for ovarian cancers, the presence of Oct-4 and other pluripotent markers in pancreatic cancers is suggestive of VSELs as the possible cancer-initiating stem cells. Several issues raised in the review require urgent confirmation and thus provide scope for further research before arriving at a consensus on the fundamental role played by VSELs in normal pancreas biology and during regeneration, aging, and cancer. In the future, such understanding may allow manipulation of endogenous VSELs to our advantage in patients with diabetes and also to treat cancer.

## Introduction

The pancreas is one of three organs (besides lung and liver) with huge regenerative ability. However, the mechanism underlying this remarkable regeneration is still shrouded in controversy and was recently reviewed [1,2]. Views are divided as to whether regeneration involves stem cells or is a mere reduplication of pre-existing islets and also whether the number of islets is fixed by birth or they are replenished possibly by the ductal epithelial (DE) cells. Understanding the basic mechanism responsible for pancreatic regeneration and whether stem cells are involved has lot of relevance given the huge global burden of diabetes.

We recently demonstrated a role of very small embryonic-like stem cells (VSELs) in mouse pancreas regeneration after partial pancreatectomy [3], in agreement with earlier studies reporting the presence of VSELs in adult pancreas [4] and their mobilization in response to streptozotocin treatment [5]. However, a careful review of the literature reveals that a study by Xiao and colleagues [6] seems to have sealed the controversy about pancreas regeneration. Their results demonstrate that stem cells have no role during regeneration and firmly support earlier findings of Melton’s group in favor of reduplication of existing islets [7] and are in agreement with the conclusions drawn by Teta and colleagues [8] that label-retaining stem cells do not exist in pancreas. These studies have also contradicted the concept proposed by Bonner-Weir and Sharma [9] that DE cells may have a role during regeneration of pancreas.

Our results that VSELs may have a role in pancreatic regeneration [3] may be disregarded with time and die a slow death because of the prevailing views in the field of pancreas biology and also because the very existence of VSELs is riddled with controversy [10]. This review is our humble attempt to make a strong case for VSELs during pancreas regeneration, aging, and carcinogenesis and to point out technical reasons that may explain why the pancreatic VSELs have eluded the scientific community until now.

### An introduction to very small embryonic-like stem cells

Readers may refer to recent publications to understand VSEL biology [11-14]. In brief, VSELs are small (3- to 5-μm) cells which can be enriched by flow cytometry as LIN^−^/CD45^−^/SCA^+^ cells in mice and as LIN^−^/CD45^−^/CD133^+^ cells in humans. It is suggested that, during early embryonic development, pluripotent primordial germ cells (PGCs) migrate to various developing organs, including the gonads, and survive as VSELs throughout life and serve as a backup pool for tissue-specific progenitors to maintain normal steady-state, are mobilized in response to injury to various organs, and are possibly the embryonic remnants resulting in cancer during adult life [15-17]. They express various pluripotent as well as PGC-specific markers and are relatively quiescent [18,19]. VSELs give rise to cells of all three germ layers in mice [20] and also in humans [21]. However, unlike pluripotent embryonic stem cells (ESCs), VSELs neither form teratoma in severe combined immunodeficiency mice nor complement a developing embryo. The mechanisms underlying their pluripotent [19] and quiescent [22,23] states have been well studied.

The very existence of VSELs was recently debated [10], and the reasons resulting in the controversy were explained [14]. Our group has focused on VSEL biology in adult testes and ovaries [24,25]. Recently, we made a case for VSELs to explain adult ovarian biology, failure, menopause, and cancer [26,27]. Besides the role of VSELs during pancreatic regeneration [3], bone marrow VSELs have recently been implicated during hepatic regeneration [28] and their vasculogenic potential has been demonstrated in patients with critical leg ischemia [29].

### Does *Oct-4* have a role in somatic tissues?

The spurt of publications reporting the presence of *Oct-4* (octamer-binding transcription factor 4) gene expression in somatic organs and cancers (Table S1 in [30]) was a cause of concern to Schöler, Jaenisch, and their group. *Oct-4* is a marker for pluripotent stem cells and is expected to be expressed in ESCs and PGCs and not in somatic tissues [31]. The group provided strong ‘ironclad’ evidence that *OCT-4* has no role in adult organ homeostasis. They used a strain of mice in which the endogenous *Oct-4* locus was targeted by lox P sites, when crossed with various strains with tissue-specific Cre recombinase, and was selectively deleted in specific organs to enable the study of organ function in the absence of *Oct-4*. They observed that ablating *Oct-4* in various somatic tissues (skin, liver, and bone marrow) had no effect on tissue homeostasis or regeneration and thus concluded that *Oct-4* has minimal effect in somatic stem cells. Berg and Goodell [32] wrote a commentary on this work and cautioned that absence of evidence is not evidence for absence. Also, they suggested that transgenic Cre recombinase possibly was expressed not in the true stem cells but only in the progenitors.

We agree with the thought process of Berg and Goodell [32], and it is likely that in the study by Lengner and colleagues [30], transgenic Cre recombinase may not have been expressed in the VSELs. Alternatively, when both VSELs and tissue-specific progenitors were deleted by the approach used by Lengner and colleagues [30], normal VSELs were mobilized from the bone marrow in response to the induced stress and brought about homeostasis. Further studies are required in this study model to help decipher the role of Oct-4 expressing VSELs in adult somatic tissues and cancers.

### The role of very small embryonic-like stem cells during pancreas regeneration

Melton’s group proposed that pancreas regeneration occurs by reduplication of pre-existing islets and that there is no role of stem cells in the process. The study by Dor and colleagues [7] had associated technical issues and was discussed [9]. A careful examination of the methods shows that five injections of tamoxifen (4 mg, intra-peritoneal or subcutaneous twice a week) were given over a period of more than 15 days and resulted in nuclear translocation of Cre estrogen receptor protein, allowing expression of human placental alkaline phosphatase (HPAP) in insulin-expressing cells and their progeny. But during this time, VSELs (expected to harbor the transgene) could also have differentiated into HPAP-expressing beta cells. Thus, it is possible that, rather than a reduplication of pre-existing islets, the new islets possibly regenerated from pluripotent VSELs in agreement with our findings [3].

Rather than using the controversial Cre system for lineage tracing, Teta and colleagues [8] used thymidine analog incorporation and found no signs of label-retaining stem cells/progenitors during adult pancreas regeneration, thus confirming the earlier findings of Dor and colleagues [7]. However, if their results are compared with our published results [3], it is apparent that they were focusing only on the islets to find label-retaining cells but we show that stem cells/progenitors occur at other distinct locations (besides the islets). Islets comprise an actively dividing and differentiating cell population (not a stem cell population) where the label may have been washed off.

Bonner-Weir’s group [33] proposed that pancreas regeneration involves DE cells which undergo de-differentiation and differentiate into islets and acinar cells. Three groups [34-36] carried out lineage-tracing studies using different DE-specific markers to investigate whether ductal cells could be the source of new beta cells and resulted in inconsistent data. Whereas Bonner-Weir’s group found direct evidence in support of neogenesis by using carbonic anhydrase II promoter as the marker for lineage tracing, others, using Sox-9 and Hnf, failed to support these results. This controversy was discussed [37], and we propose that the use of markers like Sca-1, Nanog, Sox-2, or Oct-4 for lineage tracing will provide more meaningful results.

Xiao and colleagues [6] provided strong evidence to support earlier views of Melton’s group that pancreas regeneration does not involve stem cells. The group used a tamoxifen-free technique wherein they employed a dual reporter system in which expression of Cre recombinase driven by the insulin promoter causes the deletion of a red fluorescent reporter and simultaneously activates a green fluorescent reporter. If a red non-beta cell differentiated into a beta cell (neogenesis), it would turn on the insulin gene and Cre recombinase. For a brief period, the overlap of red and green fluorescence would produce a yellow signal until the red fluorescent protein degrades and the cell turns permanently green. Using these mice, the authors observed that developing pancreas had both green and yellow cells but that later on in life and in all the models of adult beta-cell growth/regeneration (pregnancy, partial pancreatectomy, and treating pancreas with alloxan or streptozotocin and duct ligation) yellow cells were not detected by flow cytometry. Thus, the absence of yellow cells in their flow cytometry study was interpreted as the absence of neogenesis from stem cells and supported the concept of reduplication during regeneration of adult mouse pancreas. In fact, the group removed all subjectivity from their approach by using whole pancreas cell suspension and assessed the results by the quantitative approach of flow cytometry. One of the workers in the field mentioned that their study provided the final nail in the coffin for beta-cell neogenesis in adult mice pancreas [38].

We were indeed taken aback by the data generated by Xiao and colleagues [6] but the group spun their pancreas cell suspension at 1,200 revolutions per minute (rpm) while processing for flow cytometry experiments. Earlier we reported that VSELs get easily discarded during the volume reduction step while processing cord blood and bone marrow using the standard Ficoll-hypaque density gradient centrifugation method [39]. The blood stem cells get separated in the ‘buffy coat’, whereas the VSELs settle down along with red blood cells and always get discarded [39]. It has taken us a few years of experience to realize that, because of their very small size, VSELs do not pellet easily. VSELs exist in ovary surface epithelium [40] and in order to enrich them, surface epithelium of a large number of sheep ovaries was scraped almost four times a week for almost 2 months and attempts were made to pellet down the VSELs by spinning at 1,200 rpm. After a long struggle, we found the VSELs in the supernatant and successfully pelleted them by increasing the speed from 1,200 rpm to 1,000G. Now we regularly spin at 1,000G to enrich VSELs for making smears and at 3,000G for putting in TRIZOL for RNA extraction. VSELs are truly the ‘missed pearls’ as described by Mariusz Ratajczak’s group in various adult mouse tissues, including pancreas [41].

VSELs do exist in the pancreas and we are intrigued by the fact that similar confusion exists in the field of ovarian biology. Similar to Xiao and colleagues [6], Lei and Spradling [42] used the lineage-tracing approach and concluded that adult mouse ovaries lack stem cells. They proposed that primordial follicle pool generated during fetal life is sufficient to sustain oogenesis and that there is no renewal of oocytes during adult life. But we discussed the technical caveat in their study and have shown that VSELs are present in adult mouse ovaries [43]. To conclude, it is apparent that technology can never overtake biology and Mother Nature always has more force than the wisdom of the humans. Although not in the strict sense of lineage tracing, we have data to demonstrate lineage derivation of progenitors from the VSELs. A careful examination of our published results in testis [44], ovary [40], and pancreas [3] shows how pluripotent VSELs with nuclear Oct-4 give rise to tissue-committed progenitors with cytoplasmic Oct-4. Evidently, the differentiated tissue-specific progenitors (where nuclear Oct-4 is no longer required to maintain a pluripotent state) express cytoplasmic Oct-4, which is eventually lost as the cells differentiate further. There are three more fronts in favor of VSEL biology in pancreas: (i) Oct-4^+^ cells in human pancreas, (ii) effect of aging on pancreatic biology, and (iii) embryonic markers expressed in pancreatic cancer.

### Available studies on Oct-4^+^ cells in human pancreas

Oct-4^+^ cells (of two distinct sizes) have been reported in human pancreas, although they were not called VSELs. Zhao and colleagues [45] detected stem cell markers Oct-4, SOX-2, and CD34 in islet-enriched fractions of all 25 adult human pancreases, and there were no significant differences between endocrine and exocrine cell fractions. Immunohistochemical staining for Oct-4, SOX-2, CD133, CD34, CK19, insulin, and nestin on human pancreas sections showed that the majority of Oct-4^+^ cells were found in the walls of small ducts. Similar localizations were observed for SOX-2^+^ cells. The majority of SOX-2^+^ cells were found to co-express Oct-4 proteins, but not *vice versa*. The majority of Oct-4^+^ cells had cytosolic staining, whereas a small percentage (approximately 1.6%) of cells showed nuclear positivity. White and colleagues [46] found nuclear co-expression of pluripotent markers Oct-4/SOX-2/NANOG in proliferative ‘islet survivor cells’ and also in adult human pancreas as well as an islet fraction sample by reverse transcription-polymerase chain reaction. Various techniques like confocal microscopy, flow cytometry, and Western blotting were used to confirm the results. These cells are very small (1.5 to 3 μm), resembling VSELs. Expression of Oct-4 in human pancreas-derived primary cell cultures has been reported by other groups also [46,47]. These results in human pancreas, describing the presence of smaller cells with nuclear Oct-4 and slightly bigger cells with cytoplasmic Oct-4, correlate well with our findings in mice that VSELs have nuclear Oct-4 whereas slightly bigger cells express cytoplasmic Oct-4 and PDX-1 [3]. Thus, based on these studies, our findings in mice pancreas become relevant to humans as well.

The presence of Oct-4^+^ cells in the walls of the small ducts possibly explains the confusing concept put forth by Bonner-Weir’s group of the involvement of ductal epithelium in pancreas regeneration. During various studies performed by Bonner-Weir's group, VSELs were possibly resulting in a burst of proliferative activity in the vicinity of ductal epithelium during regeneration, which was mistakenly interpreted as transdifferentiation of the epithelial cells into islets.

### The effect of aging on pancreatic biology

Age-associated decline in beta-cell function is becoming apparent and explains the risk for diabetes with advanced age [48]. The majority of patients with type 2 diabetes mellitus (T2DM) are more than 40 to 50 years old [49]. Similarly, Paulson and colleagues [50] showed that the presence of gestational diabetes is increased in mothers with advanced age. Also, islets isolated from aged donors result in poor transplantation outcomes compared with young donors [51]. Kushner [48] put forth three alternative hypotheses: extended cell cycle length, fewer aged beta cells entering the cell cycle, or their limited numbers after puberty may be responsible for age-related decline. But then why does the beta-cell mass increase with obesity? As reproductive biologists, we are tempted to compare pancreas and ovarian biology. It has been proposed that menopause occurs because with advanced age the somatic microenvironment ‘niche’ is unable to support stem cell function [26]. Similarly, aged pancreas will house VSELs but they are unable to undergo differentiation because of a compromised niche and this may explain the increased incidence of T2DM with increased age.

### Embryonic markers expressed in pancreatic cancer

**Figure 1:**
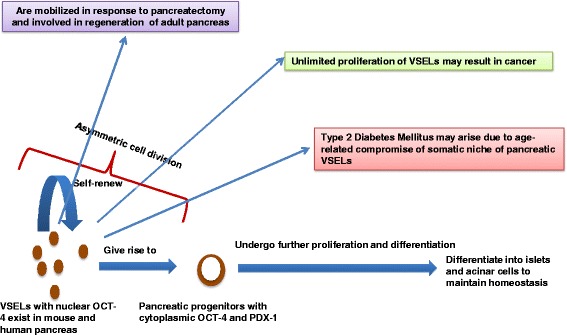
The pivotal role played by very small embryonic-like stem cells in pancreas biology. OCT-4, octamer-binding transcription factor 4; VSEL, very small embryonic-like stem cell.

Herreros-Villanueva and colleagues [52] have reported the aberrant presence of key ESC-specific markers like Oct-4, Nanog, and SOX-2 in cases of pancreatic ductal cell carcinoma. Similarly, Lu and colleagues [53] showed that knockdown of Oct-4 and Nanog expression inhibits the stemness of pancreatic cancer cells. Earlier, Wen and colleagues [54] reported expression of Oct-4 and Nanog with early stages of pancreatic carcinogenesis. All of these reports support the possibility that pluripotent VSELs, which express Oct-4, Nanog, SOX-2, Sca-1 (mice), and CD133 (humans), could be the ‘embryonic remnants’ or cancer-initiating cells in adult organs, including pancreas [15]. Starzyńska and colleagues [55] reported intensified trafficking of LIN^−^/CD45^−^/CD133^+^ VSELs and CD45^−^/CD105^+^/STRO1^+^ mesenchymal cells in patients with pancreatic cancer. Similarly, we demonstrated that VSELs are implicated during cancer initiation in various reproductive organs after neonatal endocrine disruption [27]. Similar to the concept that ovarian cancer may arise due to uncontrolled proliferation of VSELs because of a compromised niche [26], it is likely that the pancreas stem cell niche gets altered in a manner that is unable to keep the VSELs quiescent and rather they undergo uncontrolled proliferation and hence cancer (Figure 1). These rapidly dividing cancer cells will express Oct-4, which is reported in pancreatic cancer tissue by various groups as discussed above.

Besides VSELs, mesenchymal stem cells (MSCs) have been reported in the pancreas by various groups, and attempts are being made to treat type 1 diabetes and cancer with MSCs. This is a huge topic in itself and beyond the scope of the present review. But we believe that MSCs are actually the niche providing stromal cells, express cytoplasmic Oct-4, and are derived from the VSELs [56,57]. We recently restored spermatogenesis from endogenous VSELs (which survived in chemoablated mouse testis) by transplanting MSCs which provided a healthy niche [58] and allowed surviving VSELs to undergo differentiation into sperm.

## Conclusions

We have explained technical reasons why VSELs have eluded the scientific community so far and also discussed the potential role of VSELs during normal pancreas biology, regeneration, aging, and cancer (Figure 1). VSELs exist in human pancreas also. Age-related changes in the somatic microenvironment result in the decline of beta-cell regeneration and hence increased incidence of T2DM. The uncontrolled proliferation of pluripotent VSELs possibly results in cancer. Thus, our article provides an altogether new perspective and dimension to the field of pancreas biology in both mice and humans and opens up newer areas for research.

We earnestly request that others in the field carefully review their protocols for studying stem cells by flow cytometry. The use of a speed of 1,200 rpm to spin down cells, while processing them for various experiments, may explain the inability to detect VSELs in mouse bone marrow similar to while processing pancreatic cells [6,10]. Hopefully, as an outcome of this article, the controversy surrounding VSELs will settle and further studies will be undertaken to exploit their potential to regenerate the diabetic pancreas. We are intrigued by the potential of VSELs to regenerate both the islets and acinar cells in adult mouse pancreas compared with embryonic or induced pluripotent stem cells which, as reviewed recently, tend to give rise to fetal counterparts [1,59].

## Abbreviations

DE: Ductal epithelial
ESC: Embryonic stem cell
HPAP: Human placental alkaline phosphatase
MSC: Mesenchymal stem cell
Oct-4: Octamer-binding transcription factor 4
PGC: Primordial germ cell
rpm: revolutions per minute
T2DM: Type 2 diabetes mellitus
VSEL: Very small embryonic-like stem cell
